# Supplementary material for: Advanced Skeletal Ossification Is Associated with Genetic Variants in Chronologically Young Beef Heifers
Source: Genes (Basel). 2023 Aug 15;14(8):1629. doi: 10.3390/genes14081629 (PMC10454746; doi:10.3390/genes14081629)
Supplement: Supplementary file 1 [file genes-14-01629-s001.zip › genes-2551245-supplementary.pdf]

Table S1: Primer and probe sequences designed for custom TaqMan MGB Allelic Discrimination Assays. Probes are listed with the dyes used for each probe which are listed as VIC and FAM. The target is named starting with the gene name and then followed by SNP with chromosome and base pair location.

Gene	Primer	Probe
ALPL SNP_2:131837201	Forward Primer: CGTGCTAAGTCACTTCAGTCATGTC	Probe 1 (VIC): ACTCTGTGATCCTACGGA
	Reverse Primer: CCCAGATCAAAGAAAAGAGTAAGATAAAT	Probe 2 (FAM): CTGTGATCCTACAGACT
ESR1 SNP_9:90115650	Forward Primer: AAAAGTGATGGTGTCCCTTGAAA	Probe 1 (VIC): CTCAGGACGCCAGA
	Reverse Primer: CCCAAGGCTCTGTAAAAGATTAACC	Probe 2 (FAM): CTCAGGGCGCCAGAT
ESR1 SNP _9:90015095	Forward Primer: CAAGAGATGAACAAATAAACCAGCAA	Probe 1 (VIC): AGGTTGAAATCAGGTCT
	Reverse Primer: AGGTTGAAATCAGGCCT	Probe 2 (FAM): AGGTTGAAATCAGGCCT

Table S2: Custom primer designs used in targeted gene sequencing for locations. Primer target lists the gene of interested that was intended for sequencing. Some targets had two primer sets designed for it and that is denoted in the title after the gene name as "Set 1" or "Set 2".

Primer Target	Primer Pair Sequence
OSTERIX Set 1	Forward Primer: 5'-AGACAAGACACTGCTTAGGGTGAGT– 3'<br>Reverse Primer: 5'-TCTCTCTCCCACTCCTCTCTCTTCA -3'
OSTERIX Set 2	Forward Primer: 5'-GACTGCCCTCTCCTTTCTCTCATTG -3'<br>Reverse Primer: 5'-ATGTACTCACTGTTGCCCAGTGCCC -3'
COL10A1	Forward Primer: 5'-CTGAAATTCAGATTACAACATGTTT-3'<br>Reverse Primer: 5'-CAAATACGCTGACATGGAGGTCTAC-3'
PTHLH Set 1	Forward Primer: 5'-AAAAATTGGCTCTGTTCCAAGACAG-3'<br>Reverse Primer: 5'-GCTGAAGACAATTTCCAGAAATACAT-3'
PTHLH Set 2	Forward Primer: 5'-TCCTCTCCTTTAAAAGTTAGCCCTT-3'<br>Reverse Primer: 5'-AGCAGACTAATGTTTTGTCTAGCAC-3'
RUNX2 Set 1	Forward Primer: 5'-AGGGTGGTAATGAGAAAAGATAGCC-3'<br>Reverse Primer: 5'-AGCACCATAAATGCAAGAGTATAGT-3'
RUNX2 Set 2	Forward Primer: 5'-AAGTTTAGGGAGGTGGGAAAACAT-3'<br>Reverse Primer: 5'-CCATGTGTTATCTATGGTTTTGTTT-3'
RUNX2 Set 3	Forward Primer: 5'-CAAGTTTAGGGAGGTGGGAAAACAT-3'<br>Reverse Primer: 5'-CCATGTGTTATCTATGGTTTTGTTT-3'
SOX 9	Forward Primer: 5'-GCAGTTTTAAAGTTGTTTTTAGTCA-3'<br>Reverse Primer: 5'-CTCACTTTAAGTTTTTGTCTTTAAA-3'
BMP2 1	Forward Primer: 5'-ATGCCTGAAACCCTGGCTTAAGCAA-3'<br>Reverse Primer: 5'-CATGCCTTTTGCCCTCATCTTCCCC-3'
ESR2 Set 1	Forward Primer: 5'-TCCACAGTGGCTGTGTCATT – 3'<br>Reverse Primer: 5'-GACCTATGACCTCTGTCCATCAA -3'
ESR2 Set 2	Forward Primer: 5'-GAGACCCCAGTTCGATTCCT -3'<br>Reverse Primer: 5'-GACTCTGTGACCCCATGGAC -3'
GHR	Forward Primer: 5'-ATCCCTCCTCCAACCCTCTC-3'<br>Reverse Primer: 5'-AGCAACAAGGATGCAACTAAAGA-3'

Table S3. Summary of SNPs that were identified in target genes and were not found to be significant.

Target Gene	No. SNPs
GHR	13
PTHLH	1
RUNX2	2
SP7 (Osterix)	1
